# The posterior-anterior-flexed view is essential for the evaluation of valgus osteoarthritis. A prospective study on 134 valgus knees

**DOI:** 10.1186/s12891-019-3012-3

**Published:** 2019-12-30

**Authors:** Kilian Rueckl, Armin Runer, Ulrich Bechler, Martin Faschingbauer, Sebastian Philipp Boelch, Peter Keyes Sculco, Friedrich Boettner

**Affiliations:** 1 Hospital for Special Surgery, 535 East 70th Street, New York, NY 10021 USA; 2 Department for Orthopedic Surgery, Koenig-Ludwig-Haus, University of Wuerzburg, Brettreichstrasse 11, 97074 Wuerzburg, Germany; 3 Department for Orthopedic Surgery, University of Ulm, Oberer Eselsberg 45, D-89081 Ulm, Germany

**Keywords:** Valgus osteoarthritis, Knee, PA-flexed view, View, Radiographs

## Abstract

**Background:**

Radiographic imaging is an important tool to assess osteoarthritis (OA). Lateral compartment osteoarthritis (valgus OA) usually starts with cartilage degeneration along the posterior aspect of the lateral femoral condyle. There is evidence that the posterior-anterior (PA)-flexed view is more sensitive when diagnosing early stages of valgus OA compared to the anterior-posterior (AP) view. The current paper analyzes the value of the PA-flexed view for patients scheduled for total knee arthroplasty (TKA).

**Methods:**

Radiographs of 134 valgus knees were assessed prior to TKA. The minimal joint space width (minJSW) was measured on AP and PA-flexed views. The extent of mechanical deformity was measured on hip to ankle standing films.

**Results:**

49 (36.6%) AP views showed Kellgren and Lawrence (K/L)-grade 4 osteoarthritis in the lateral compartment, 82 (63.4%) showed grade 3 or less. The PA-flexed view resulted in an increased K/L-grading to grade 4 for 53 knees (62.4%) that were considered grade 3 or less on standard AP-radiographs. There was a significant differences between lateral minJSW on AP and PA-flexed view for patients with up to 10 degrees of mechanical valgus deformity (*p* < 0.001), as well as 11 to 15 degrees of mechanical deformity (*p* = 0.021). Only knees with severe deformity of more than 15 degrees did not show a difference in minJSW between PA-flexed view and AP view (*p* = 0.345).

**Conclusions:**

The PA-flexed view is superior to the standard AP view in quantifying the extent of valgus OA in patients with zero to fifteen degrees of valgus deformity. It is recommended for the initial assessment of patients with valgus osteoarthritis and better documents the extent of osteoarthritis prior to TKA.

## Background

Early stages of valgus osteoarthritis (OA) mainly effect the posterior aspect of the lateral femoral condyle [1]. Weight-bearing anterior-to-posterior radiographs of the fully-extended knee (AP view) are the most common tool to diagnose OA of the knee. Widely accepted classifications for OA including the Kellgren and Lawrence (K/L) classification, the Ahlbaeck- and the osteoarthritis society international (OARSI) classification, are based on minimal joint space width (minJSW) and size of osteophytes on the AP view [2–4]. However, the AP view displays the distal aspect of the femoral condyle and may therefore underestimate the severity of valgus OA. The weight-bearing fixed-flexion posterior anterior radiographic imaging of the knee (PA-flexed view) is a supplemental radiographic image that focuses on the cartilage loss over the posterior lateral femoral condyle and has been documented to be more sensitive for the diagnosis of early valgus OA [5]. However, it is unclear if the PA-flexed view is beneficial for more advanced valgus OA and patients with advanced valgus deformity prior to total knee arthroplasty (TKA).

The current study investigates the following research questions: Is the PA-flexed view more accurate for the (K/L) grading of valgus (OA)? Does the PA-flexed view effect the minJSW measurements regardless of the grading of (OA) and extent of mechanical deformity?

## Methods

Between 2008 and 2013, the senior author performed 214 TKAs in 174 patients (171 women (138, 79.3% knees), 43 men (36, 20.7% knees), 40 (18.7%) bilateral TKAs (6 men (12 knees), 19 women (38 knees)). For 153 knees a complete set of (1) standard weight bearing AP, (2) PA-flexed-radiograph, (3) weight bearing hip-to-ankle (HA) standing radiograph and (4) merchant view were available. The following patients were excluded: (1) 2 (1.3%) patients with advanced OA with knee subluxation, (2) 16 (7.5%) patients with malrotation or malalignment of the AP or PA-flexed view with an overlap of the anterior and posterior edge of the tibial plateau of more than 3 mm as well as (3) 1 patient (0.5%) with prior osteotomy realignment surgery. 134 (87.6%) TKAs in 100 patients (77 (77.0%) women (106 knees) and 23 (23,0%) men (28 knees)) were enrolled in the current study. The mean age at time of surgery was 67.0 years (range: 40–89 years, SD 9.6). The mean BMI was 30.5 kg/m2 (range: 19.0–58.7 kg/m2, SD 7.2).

### Radiographic protocol

Radiographic protocols and measurements were performed as described before [5]. In brief, standardized AP-, PA-flexed-, merchant- and HA-radiographs were available for each knee. Knees were grouped by the extent of mechanical valgus deformity (< 5.0 deg., 5.0–9.9 deg., 10.0–14.9 deg., ≥15.0 deg.) as previously described [6]. The minJSW was measured for the medial and the lateral compartment in the AP- and PA-flexed-radiographs with digital templating software (Sectra AB, Linköping, Sweden) as previously described [5, 6]. The medial, lateral and patellofemoral compartment were graded according to the K/L classification system (2). Inter–observer reliability and intra-observer correlation were 0.96–1.00 and 0.77–0.95, respectively [5].

### Statistical analysis

Variables were depicted as means and ranges. All variables were evaluated for normal distribution with either the Kolmogorov-Smirnov or Shapiro-Wilk test. Means were tested for homogeneity of variance with the Levene test. Comparison of means or medians was done with the Wilcoxon signed rank test in case of dependent, nonparametric values and with the paired t test for dependent, parametric values, respectively. Level of significance was set at *p* < 0.05 and of high significance at *p* < 0.01. Power calculation revealed a sample size of 58 knees for a power of 80% with G*Power, version 3.1.9.2. Statistic calculations were performed with IBM SPSS® version 25.0.0.0 (SPSS, Chicago, USA).

## Results

The mean preoperative alignment of the enrolled 134 knees was 8.9 deg. of mechanical (range: 0.4–29.7 deg., SD 5.3) and 14.3 deg. of anatomical valgus deformity (range: − 2.0–34.8 deg., SD 5.5) respectively. The K/L-score on the AP view was grade 1 in none, grade 2 in 5 (3.7%), grade 3 in 80 (59.7%) and grade 4 in 49 (36.6%) knees. The K/L-score on PA-flexed view was grade 1 in none, grade 2 in 1 (0.7%), grade 3 in 31 (23.1%) and grade 4 in 102 (76.1%) knees.

**Fig. 1:**
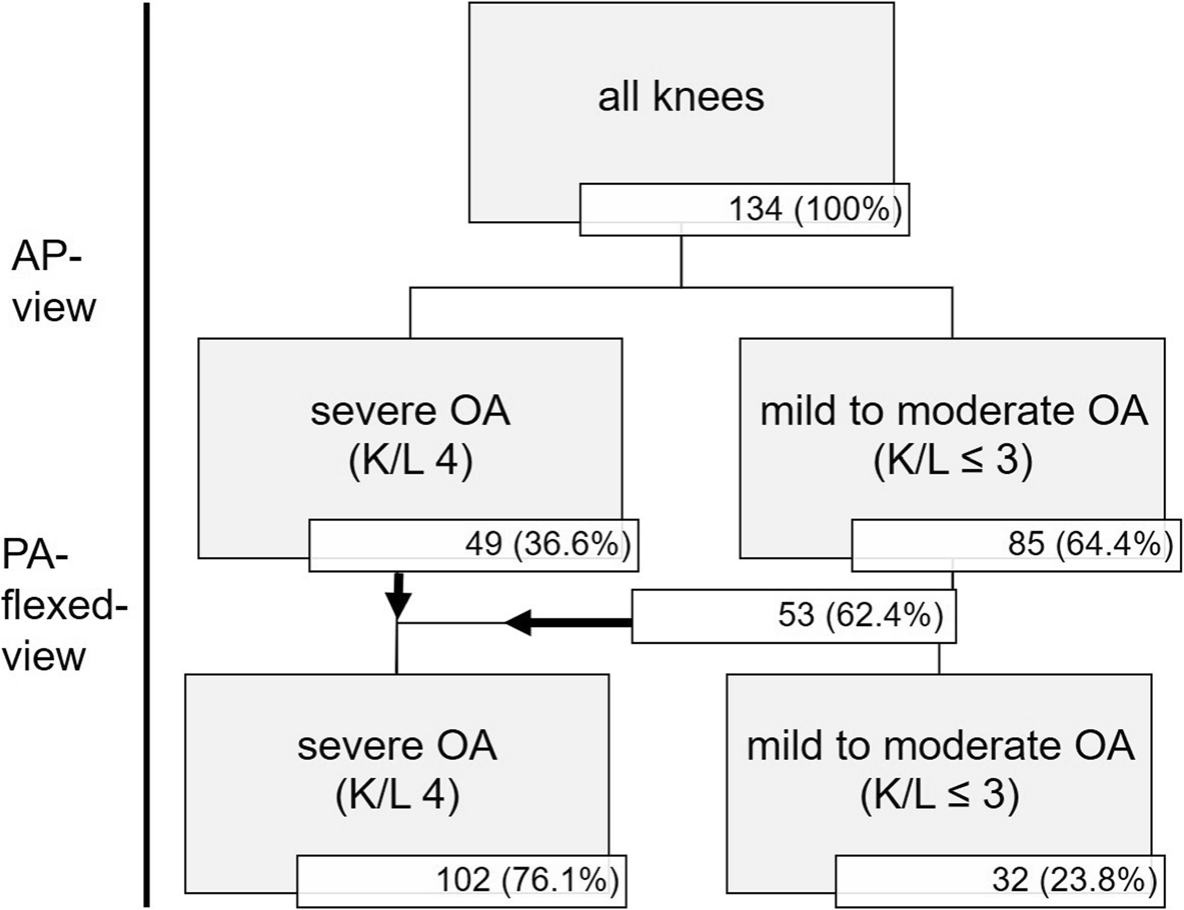
Lateral minJSW in mm for the AP- and PA-flexed-radiographs in 134 valgus knees. 76.1% of the knee showed “bone on bone” joint space narrowing on the PA-flexed view compared to only 36.6% on the AP view. The additional use of a PA-flexed view increased the OA grading level in 53 of 85 knees (62.4%)

In 53 (62.4%) of 85 knees with mild to moderate OA on AP-radiographs (K/L-score ≤ 3), the lateral femorotibial minJSW on the PA-flexed view (2.0 mm, SD 1.1) decreased highly significant (*p* < 0.001) compared to the AP view (0.1 mm) and resulted in an increased K/L-score of 4 (Fig. 1).

The mean medial minJSW was 5.5 mm (range: 0.0–12.5 mm, SD 2.1) for the AP view and 4.8 mm (range: 0.0–1.1 mm, SD 2.0) for the PA-flexed view. The mean lateral minJSW was 1.6 mm (range: 0.0–7.1 mm, SD 1.8) for the AP and 0.6 mm (range: 0.0–6.1 mm, SD 1.3) for the PA-flexed view.

In a subgroup of 68 (50.7%) knees with mild to moderate OA on the AP view (K/L-score ≤ 3) and a lateral minJSW> 1 mm, 8 (11.8%) knees had a lateral minJSW of less than 1 mm and 38 (55.9%) had “bone on bone” OA in the lateral compartment on the PA-flexed view (i.e. 0 mm lateral minJSW).

In a subgroup of 23 (17.2%) knees with mild to moderate OA in the AP view (K/L-score ≤ 3) and a lateral minJSW> 3 mm, 10 (43.5%) knees showed “bone on bone” OA in the lateral compartment and 12 knees (52.2%) were classified as K/L 4 in the PA-flexed view.

**Fig. 2:**
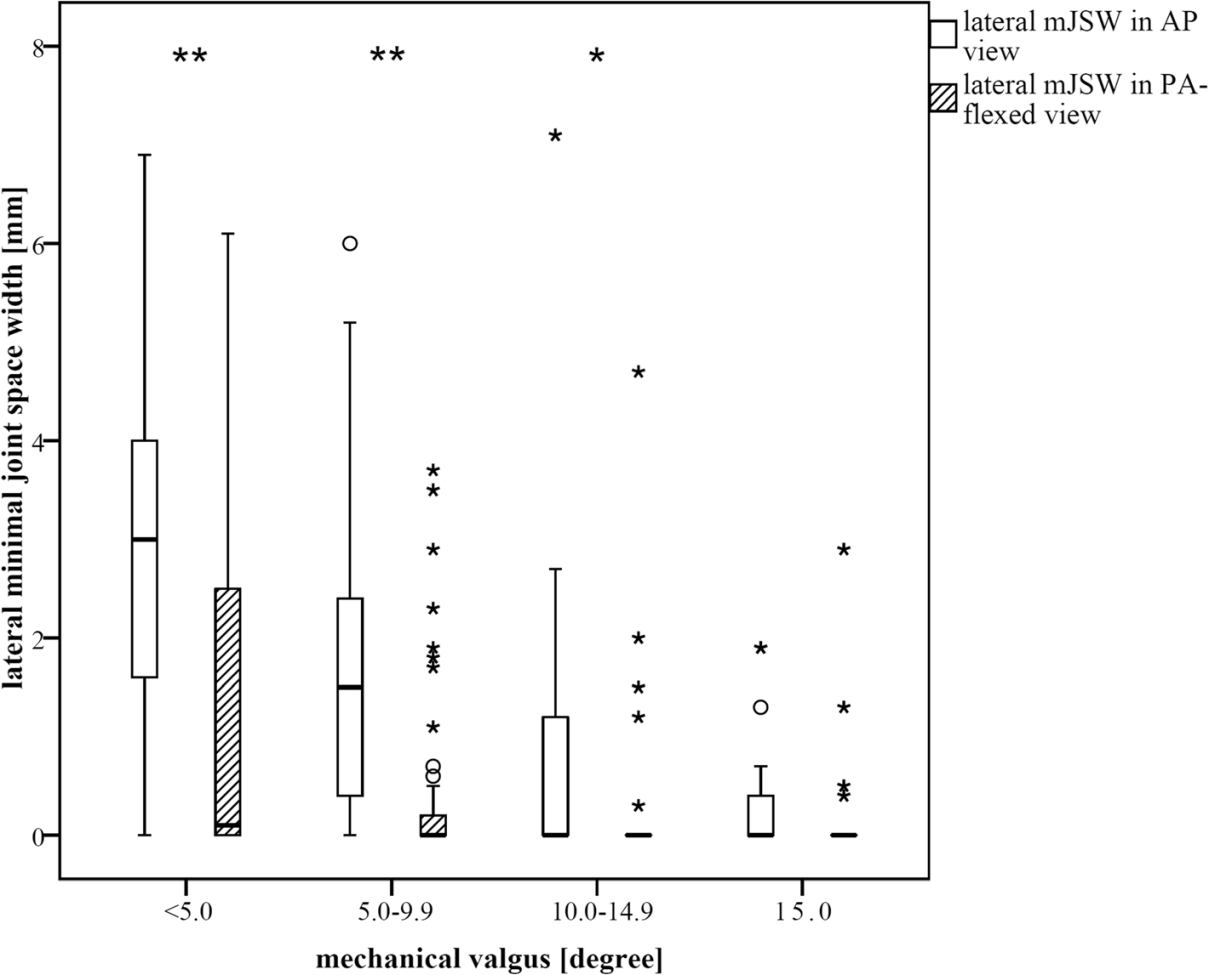
Lateral minJSW in mm on AP- and PA-flexed-radiographs in relation to the degree of mechanical alignment. Knees were grouped by the extent of mechanical valgus deformity (< 5.0 deg., 5.0–9.9 deg., 10.0–14.9 deg., ≥15.0 deg.). The most significant benefit for the PA-flexed view was in patients with less mechanical deformity. Significance-levels are marked as “*” for *p* < 0.05 and “**” for *p* < 0.01

**Fig. 3:**
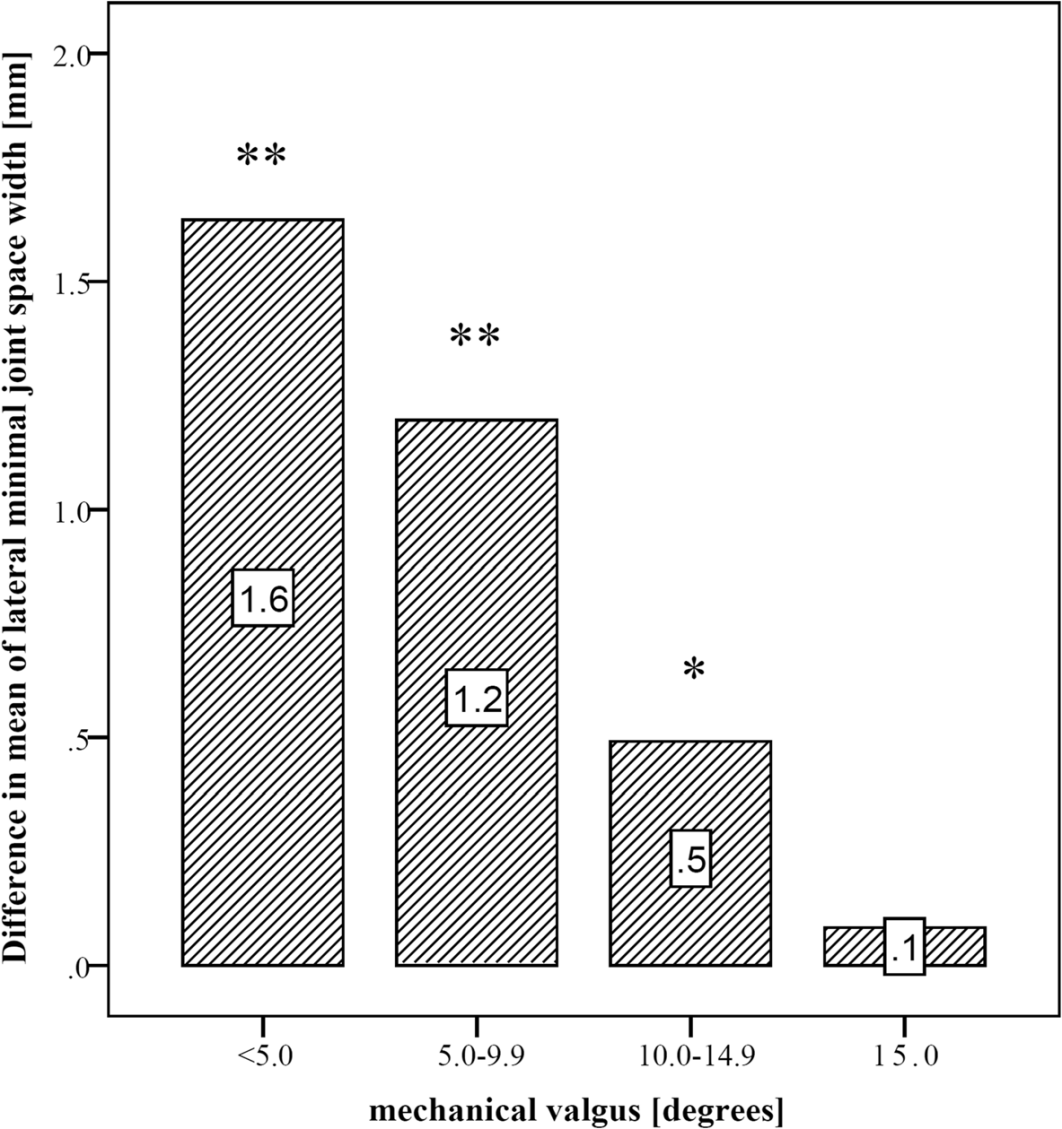
Difference in means between lateral minJSW on the AP and PA-flexed view for different groups of valgus deformity. There was a relevant and highly significant difference of minJSW in knees with mild or moderate deformity. For knees with more than 10 deg. valgus deformity the difference was significant but small (0.5 mm) or not significant at all (≥15 deg.). Significance-levels are marked as “*” for *p* < 0.05 and “**” for *p* < 0.01

**Fig. 4:**
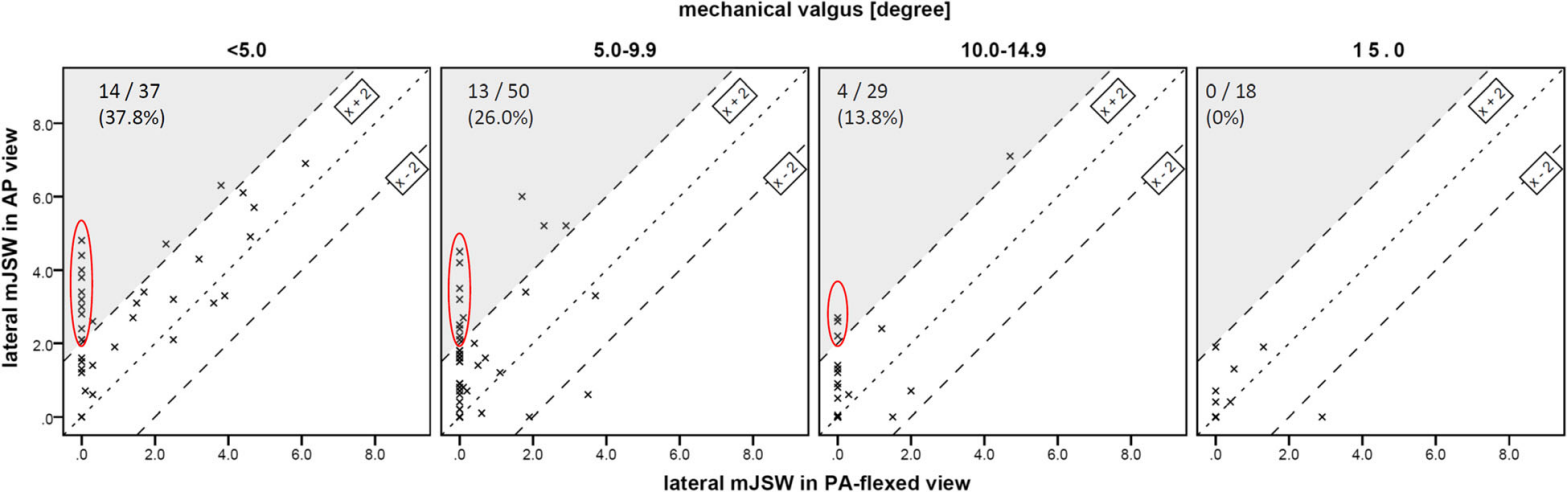
Lateral minJSW in AP and PA-flexed view for different severities of valgus deformity (< 5.0 deg., 5.0–9.9 deg., 10.0–14.9 deg., ≥15.0 deg.). The grey field mark the section where minJSW is more than 2 mm less in PA-flexed view compared to the AP view. The numbers in the upper-left display the percentages of knees in this grey field. Especially in knees with zero to ten degrees of valgus there is a substantial number of knees (37.8% or 26.0%) that had more than 2 mm minJSW in AP view but showed “bone on bone” arthritis on the PA-flexed view (red circle)

When comparing knees with different extent of mechanical valgus deformity (< 5.0 deg., 5.0–9.9 deg., 10.0–14.9 deg., ≥15.0 deg.), the difference between the lateral minJSW on AP and PA-flexed view was highly significantly (*p* < 0.001) for knees with up to 10 deg. of deformity, and significantly different (*p* = 0.021) for knees with 10.0–14.9 deg. There was no difference for knee with greater than 15 deg. deformity (*p* = 0.345) (Figs. 2, 3, 4).

**Fig. 5:**
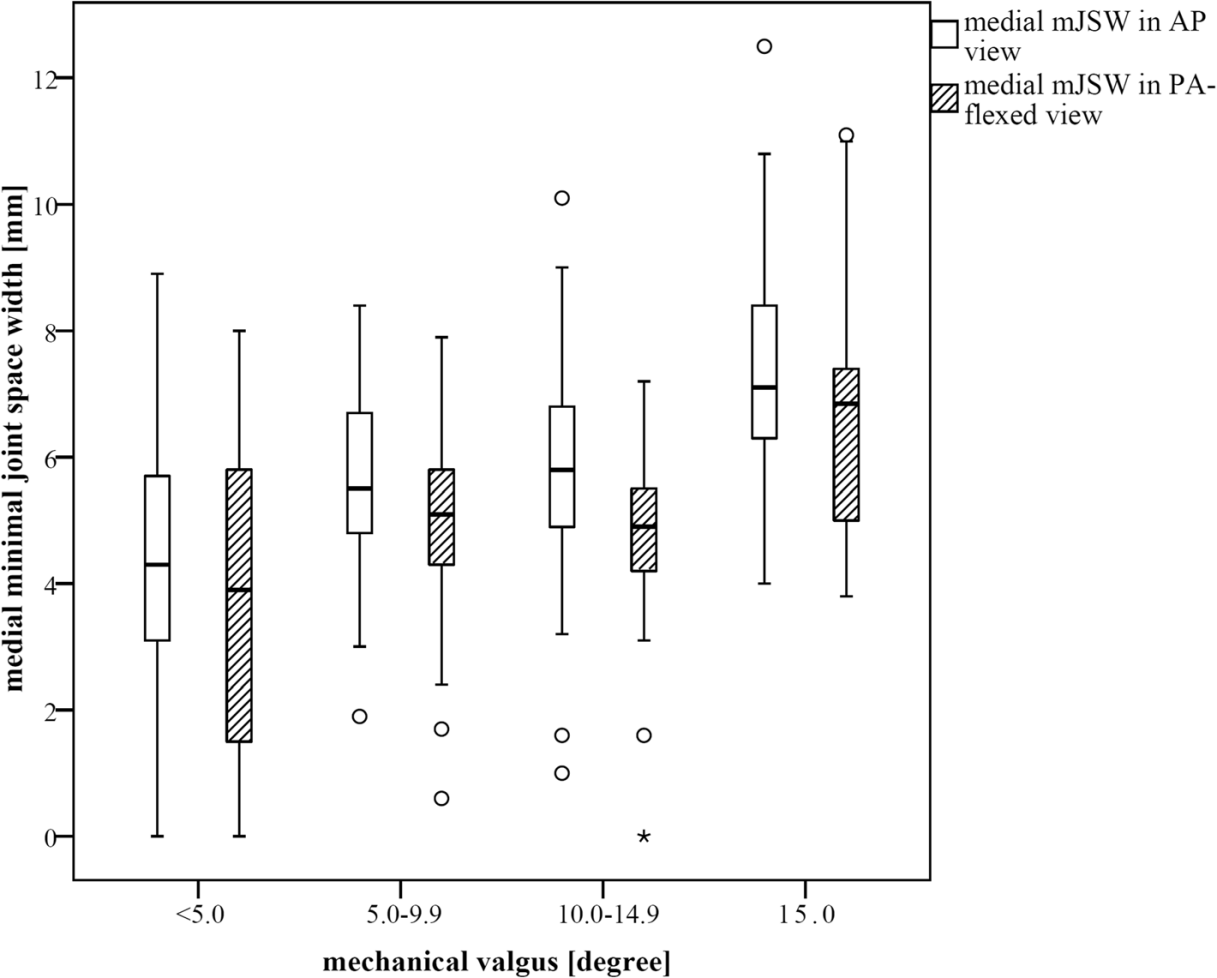
Medial minJSW in mm for AP- and PA-flexed-radiographs for different groups of mechanical valgus deformity (< 5.0 deg., 5.0–9.9 deg., 10.0–14.9 deg., ≥15.0 deg.). There was no significant difference in medial minJSW for different valgus deformities

In the medial compartment the minJSW was slightly less (Δ 0.71 mm, *p* < 0.001) on the AP view (5.5 mm, SD 2.1) compared to PA-flexed view (4.8 mm, SD2.0), with comparable differences in subgroups of different severities of valgus deformity (Fig. 5).

The extent of mechanical deformity correlated moderately with the grade of clinical medial colleteral ligament (MCL) -instability (rho = 0.49, *p* < 0.001), the degree of lateral and medial minJSW on the AP view (rho = − 0.57, *p* < 0.001; rho = 0.436, *p* < 0.001) and PA-flexed view (rho = − 0.28, *p* = 0.002; 0.30, *p* < 0.001). The grade of MCL-laxity correlated weakly with the medial minJSW on the AP view (rho = 0.302, *p* = 0.001) but not on PA-flexed view (rho = 0.170, *p* = 0.071). Correlation was comparable for the lateral minJSW on AP view (rho = 0.32, *p* = 0.001) and on PA-flexed view (rho = − 0.21, *p* = 0.022).

## Discussion

The current paper underlines the clinical benefit of PA-flexed-radiographs for assessment of valgus OA of the knee in patients with up to 15 deg. of mechanical deformity. Before surgery the PA-flexed view shows more significant joint space narrowing and K/L grading for knees with up to 15 deg. of mechanical valgus alignment.

The extend of OA on radiographs is an important criterion when indicating TKA [7]. Standard AP-radiographs have been an accepted diagnostic tool to evaluate OA [2]. minJSW and size of osteophytes allow for grading of the severity of osteoarthritis [2, 3]. In the American insurance landscape, the guideline CG-SURG-54 lists indication criteria for TKA [8]. It demands all of the following 4 criteria: (1) disabling pain; (2) failed non-operative treatment over a 3 months period; (3) limited knee function secondary to OA with the disease interfering with the ability to carry out age-appropriate activities of daily living and (4) radiographic evidence of significant joint destruction and cartilage loss (“bone on bone” OA).

However, evaluation of cartilage damage using magnetic resonance imaging (MRI) or computed tomography (CT) as well as arthroscopic inspection questioned the reliability of two-dimensional radiographs to assess OA [9–12]. The PA-flexed view was suggested to be more sensitive, especially for early onset valgus OA which is often located on the more posterior aspect of the femoral condyle [13, 14]. A recent publication from our high volume institution showed more significant joint space narrowing on the PA-flexed view in 68% of knees with mild valgus OA and similar accuracy compared to AP radiographs in varus OA [5]. The current study suggests that the PA-flexed view is beneficial for the assessment of valgus OA with up to 15 deg. of mechanical deformity. Only in knees with severe deformities of 15 deg. or more AP and PA-flexed view show comparable results (Fig. 3). The study therefore suggests that the PA-flexed view should be the preferred imaging technique in all patients with valgus OA.

AP view lack radiologic evidence of OA in patients with a more regional cartilage loss. Ultimately, this may delay surgical treatment and leave patients with failed non-operative treatment unsatisfied and underserved.

The PA-flexed view is performed in about 30 deg. of flexion of the knee [15]. This is the classic position to evaluate medial laxity in valgus OA [16]. As the PA-flexed view is a weight-bearing technique, there could be a possible utilization as a valgus stress view to detect the laxity of the MCL in valgus knees [17]. However, in our series, minJSW measurements for the medial compartment showed no differences between AP and PA-flexed view (*p* < 0.001) (Fig. 5). There is no correlation of the medial joint space width and the clinically extent of MCL laxity. One reasonable explanation might be that, when performing the PA-flexed view, the patient is advised to lean his tights against the film. This may stabilize the leg along the transversal axis and neutralize the valgus stress to the knee joint. It seems that the PA-flexed view has no benefit over the AP view in the assessment of MCL laxity.

The current study has the following limitations: (1) minJSW was measured manually in digital radiographs. Manual measurements are slightly less accurate (SD 0.1 mm to 0.2 mm) [6, 18, 19] and reproducible [20] compared to automatic measurements. However, automatic measurements are not readily available in practice and manual measurement of the minJSW are more commonly used [14, 18]. (2) Mal-rotation or skewed images can affect the accuracy of the measurement and alignment of the x-ray beam with the tibia plateau has an impact on apparent minJSW [21]. Overlapping of the anterior and posterior boarder of the medial tibia plateau also affects minJSW (1.0 mm overlap can lead to 1.0 mm error in minJSW), especially in the medial compartment [22]. As a baseline, Vignon and Brandt et al. suggested an inter-margin-distance of up to 1.5 mm as evidence of satisfactory parallel alignment [23]. Using fluoroscopy to avoid malalignment, as described by Buckland-Wright and Macfarlane et al. [6], did not provide better results [18]. With its convex configuration, we believe the lateral minJSW is less affected by overlapped radiographs. In our series, we excluded patients with an inter-margin distance of more than 3.0 mm as well as mal-rotated radiographs. (3) This paper specifically investigated OA in knees with valgus deformity. Its conclusions do not apply to knees with neutral or varus mechanical alignment.

## Conclusions

The current paper suggests that the PA-flexed view is superior to the standard AP view in quantifying the extent of joint space narrowing in valgus OA in patients with zero to fifteen deg. of mechanical deformity. The PA-flexed view appears to be beneficial during the initial and pre-surgical imaging since it better shows the true extent of degenerative cartilage loss.

## Data Availability

All data can be requested at k-rueckl.klh@uni-wuerzburg.de.

## Abbreviations

AP view: Weight-bearing anterior-to-posterior radiographs of the fully-extended knee
AP: Anterior-posterior
CT: Computed tomography
HA: Hip-to-ankle
ICC: Intraclass correlation coefficient
IRB: Institutional review board
K/L: Kellgren and Lawrence
MCL: Medial collateral ligament
minJSW: Minimal joint space width
MRI: Magnetic resonance imaging
OA: Osteoarthritis
OARSI: Osteoarthritis society international
PA-flexed view: Weight-bearing fixed-flexion posterior anterior radiographic imaging of the knee
PA: Posterior-anterior
TKA: Total knee arthroplasty
